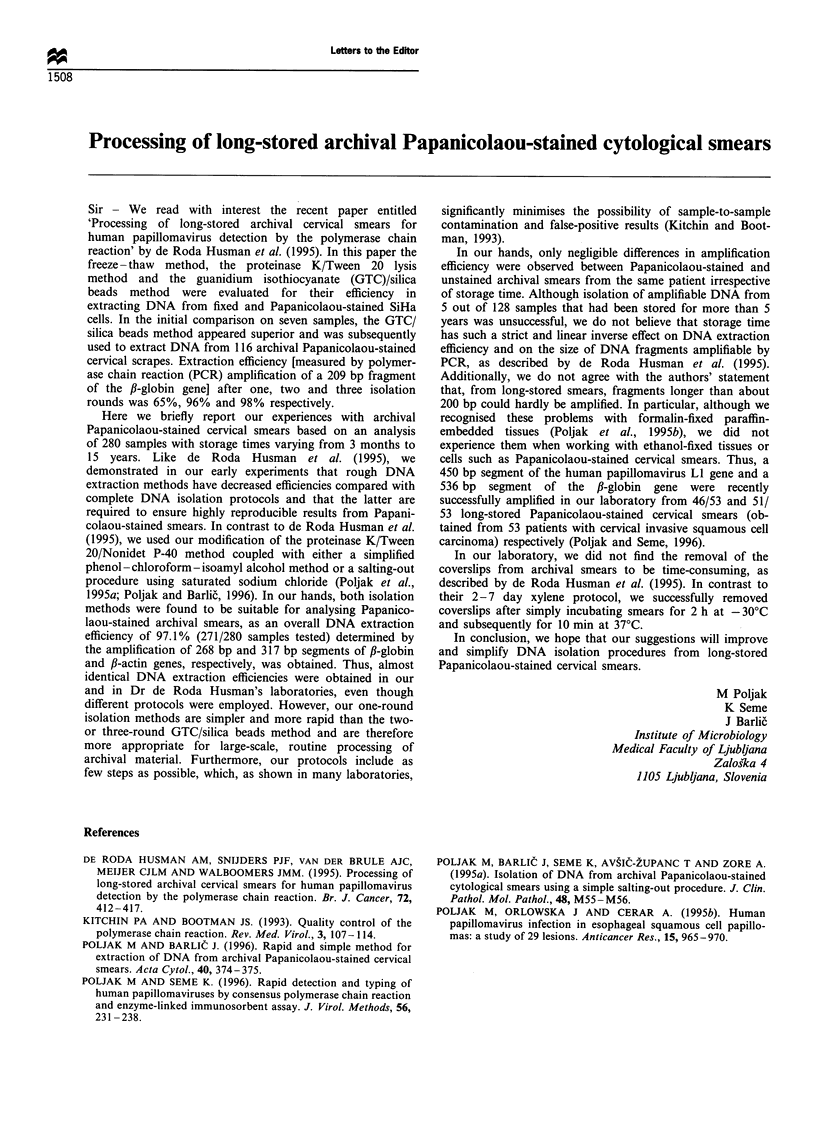# Processing of long-stored archival Papanicolaou-stained cytological smears.

**DOI:** 10.1038/bjc.1996.576

**Published:** 1996-11

**Authors:** M. Poljak, K. Seme, J. Barlic


### Processing of long-stored archival Papanicolaou-stained cytological smears

Sir – We read with interest the recent paper entitled 'Processing of long-stored archival cervical smears for human papillomavirus detection by the polymerase chain reaction' by de Roda Husman *et al.* (1995). In this paper the freeze–thaw method, the proteinase K/Tween 20 lysis method and the guanidium isothiocyanate (GTC)/silica beads method were evaluated for their efficiency in extracting DNA from fixed and Papanicolaou-stained SiHa cells. In the initial comparison on seven samples, the GTC/silica beads method appeared superior and was subsequently used to extract DNA from 116 archival Papanicolaou-stained cervical scrapes. Extraction efficiency [measured by polymerase chain reaction (PCR) amplification of a 209 bp fragment of the $\beta$-globin gene] after one, two and three isolation rounds was 65%, 96% and 98% respectively.

Here we briefly report our experiences with archival Papanicolaou-stained cervical smears based on an analysis of 280 samples with storage times varying from 3 months to 15 years. Like de Roda Husman *et al.* (1995), we demonstrated in our early experiments that rough DNA extraction methods have decreased efficiencies compared with complete DNA isolation protocols and that the latter are required to ensure highly reproducible results from Papanicolaou-stained smears. In contrast to de Roda Husman *et al.* (1995), we used our modification of the proteinase K/Tween 20/Nonidet P-40 method coupled with either a simplified phenol–chloroform–isoamyl alcohol method or a salting-out procedure using saturated sodium chloride (Poljak *et al.*, 1995*a*; Poljak and Barlič, 1996). In our hands, both isolation methods were found to be suitable for analysing Papanicolaou-stained archival smears, as an overall DNA extraction efficiency of 97.1% (271/280 samples tested) determined by the amplification of 268 bp and 317 bp segments of $\beta$-globin and $\beta$-actin genes, respectively, was obtained. Thus, almost identical DNA extraction efficiencies were obtained in our and in Dr de Roda Husman's laboratories, even though different protocols were employed. However, our one-round isolation methods are simpler and more rapid than the two- or three-round GTC/silica beads method and are therefore more appropriate for large-scale, routine processing of archival material. Furthermore, our protocols include as few steps as possible, which, as shown in many laboratories, significantly minimises the possibility of sample-to-sample contamination and false-positive results (Kitchin and Bootman, 1993).

In our hands, only negligible differences in amplification efficiency were observed between Papanicolaou-stained and unstained archival smears from the same patient irrespective of storage time. Although isolation of amplifiable DNA from 5 out of 128 samples that had been stored for more than 5 years was unsuccessful, we do not believe that storage time has such a strict and linear inverse effect on DNA extraction efficiency and on the size of DNA fragments amplifiable by PCR, as described by de Roda Husman *et al.* (1995). Additionally, we do not agree with the authors' statement that, from long-stored smears, fragments longer than about 200 bp could hardly be amplified. In particular, although we recognised these problems with formalin-fixed paraffin-embedded tissues (Poljak *et al.*, 1995*b*), we did not experience them when working with ethanol-fixed tissues or cells such as Papanicolaou-stained cervical smears. Thus, a 450 bp segment of the human papillomavirus L1 gene and a 536 bp segment of the $\beta$-globin gene were recently successfully amplified in our laboratory from 46/53 and 51/53 long-stored Papanicolaou-stained cervical smears (obtained from 53 patients with cervical invasive squamous cell carcinoma) respectively (Poljak and Seme, 1996).

In our laboratory, we did not find the removal of the coverslips from archival smears to be time-consuming, as described by de Roda Husman *et al.* (1995). In contrast to their 2–7 day xylene protocol, we successfully removed coverslips after simply incubating smears for 2 h at −30°C and subsequently for 10 min at 37°C.

In conclusion, we hope that our suggestions will improve and simplify DNA isolation procedures from long-stored Papanicolaou-stained cervical smears.

M Poljak
K Seme
J Barlič
*Institute of Microbiology*
*Medical Faculty of Ljubljana*
*Zaloška 4*
*1105 Ljubljana, Slovenia*

### References

DE RODA HUSMAN AM, SNIJDERS PJF, VAN DER BRULE AJC, MEIJER CJLM AND WALBOOMERS JMM. (1995). Processing of long-stored archival cervical smears for human papillomavirus detection by the polymerase chain reaction. *Br. J. Cancer*, **72**, 412–417.

KITCHIN PA AND BOOTMAN JS. (1993). Quality control of the polymerase chain reaction. *Rev. Med. Virol.*, **3**, 107–114.

POLJAK M AND BARLIČ J. (1996). Rapid and simple method for extraction of DNA from archival Papanicolaou-stained cervical smears. *Acta Cytol.*, **40**, 374–375.

POLJAK M AND SEME K. (1996). Rapid detection and typing of human papillomaviruses by consensus polymerase chain reaction and enzyme-linked immunosorbent assay. *J. Virol. Methods*, **56**, 231–238.

POLJAK M, BARLIČ J, SEME K, AVŠIČ-ŽUPANC T AND ZORE A. (1995*a*). Isolation of DNA from archival Papanicolaou-stained cytological smears using a simple salting-out procedure. *J. Clin. Pathol. Mol. Pathol.*, **48**, M55–M56.

POLJAK M, ORLOWSKA J AND CERAR A. (1995*b*). Human papillomavirus infection in esophageal squamous cell papillomas: a study of 29 lesions. *Anticancer Res.*, **15**, 965–970.